# Inlay-Retained Fixed Dental Prosthesis: A Clinical Option Using Monolithic Zirconia

**DOI:** 10.1155/2014/629786

**Published:** 2014-05-21

**Authors:** Davide Augusti, Gabriele Augusti, Andrea Borgonovo, Massimo Amato, Dino Re

**Affiliations:** ^1^Department of Oral Rehabilitation, Istituto Stomatologico Italiano, University of Milan, Milan, Italy; ^2^Department of Oral Surgery, Dental Clinic, Ospedale maggiore Policlinico, Fondazione IRCCS Ca' Granda, Milan, Italy; ^3^Department of Medicine and Surgery, University of Salerno, Salerno, Italy

## Abstract

Different indirect restorations to replace a single missing tooth in the posterior region are available in dentistry: traditional full-coverage fixed dental prostheses (FDPs), implant-supported crowns (ISC), and inlay-retained FDPs (IRFDP). Resin bonded FDPs represent a minimally invasive procedure; preexisting fillings can minimize tooth structure removal and give retention to the IRFDP, transforming it into an ultraconservative option. New high strength zirconia ceramics, with their stiffness and high mechanical properties, could be considered a right choice for an IRFDP rehabilitation. The case report presented describes an IRFDP treatment using a CAD/CAM monolithic zirconia IRFDP; clinical and laboratory steps are illustrated, according to the most recent scientific protocols. Adhesive procedures are focused on the Y-TZP and tooth substrate conditioning methods. Nice esthetic and functional integration of indirect restoration at two-year follow-up confirmed the success of this conservative approach.

## 1. Introduction

The availability of new treatments or technologies in dentistry has two consequences: on one side it expands the range of therapies given to patients and on the other hand it stimulates the development of decision-making algorithms for specific medical conditions [1, 2].

Different indirect restorations to replace a single missing tooth in the posterior region are available in dentistry: traditional full-coverage fixed dental prostheses (FDPs), implant-supported crowns (ISC), and inlay-retained FDPs (IRFDP) [3–5]. The last one is considered a less time and expensive solution compared to the others. Resin bonded FDPs represent a minimally invasive procedure; preexisting fillings can minimize tooth structure removal and give retention to the IRFDP, transforming it into an ultraconservative option [6]. In fact, it has been demonstrated that a high amount of coronal dentin is lost during the prosthetic preparations of abutments for conventional full-coverage FDPs with an overall calculated tooth substance removal of 63% to 73% [7].

Historically, cast resin bonded FDPs were produced exclusively using noble metals like high-gold alloys; nowadays a wide range of new materials are available: hybrid microfilled or fiber-reinforced composites (FRC), ceramics with a high content of glass particles (i.e., lithium disilicate, glass-infiltrated zirconia. or alumina), or high strength ceramics (densely sintered zirconia/alumina polycrystal) to be used as frameworks for subsequent veneering or to fabricate monolithic restorations [8, 9]. All-ceramic restorations offer an excellent optical behavior promoting biomimetic integration and their surfaces showed minimal plaque accumulation when exposed intraorally [10].

During clinical function, dental restorations are subjected to biting and chewing forces; stress applied during mastication may range between 441 and 981 N in the molar region. According to DIN standards and to some authors, FDPs should withstand occlusal forces of more than 1000 N in a static fracture resistance test [11].

New high strength ceramics, with their stiffness and high mechanical properties (i.e., resistance to fracture and/or fatigue), could be considered a right choice in an IRFDP rehabilitation [12].

New zirconia color infiltration techniques can improve the color matching when monolithic restorations were planned [13].

Zirconia still presents a challenge when used with adhesive techniques due to their single-phase tetragonal crystalline structure that is not etchable by commonly used agents such as hydrofluoric acid. Debonding of the adhesive interface and delimitation and microcracks of the ceramic veneering material were the most long term failures observed and reported [14–16].

A correct FDP and tooth cavity surfaces conditioning before adhesive cementation procedures is necessary to avoid mechanical and biological complications [17, 18].

## 2. Diagnosis and Treatment Planning

A 52-year-old patient referred to the Department of Oral Rehabilitation (Istituto Stomatologico Italiano, University of Milan) with a need for a 3-unit FDP.

The patient rejected any implant therapy planned with a previous reconstructive surgery procedure (major sinus lift).

**Figure 1:**
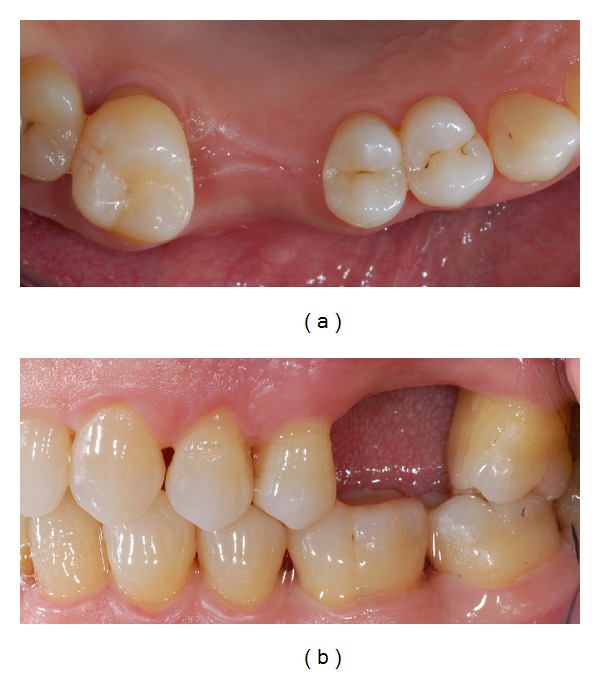
(a) Intraoral occlusal view of edentulous area before treatment. (b) Intraoral lateral view of tooth gap; the interabutment distance measured was 11 mm.

Good oral hygiene, low susceptibility to caries, coronal height over 5 mm, parallel abutments previously restored with composite fillings, and a mesiodistal edentulous gap of 11 mm were suggested for an IRFDP rehabilitation, with a minimally invasive approach compared to conventional retained full-coverage FDP (Figures 1(a) and 1(b)).

The bone level of the vital abutment teeth was radiologically investigated; no signs of active bone resorption or any periodontal and periapical pathology was revealed. The maximum mobility of grade 1 for the element 1.7 was considered acceptable; no marginal leakage, discoloration, or secondary caries of the previous composite restorations were clinically detected.

Informed consent was obtained from the patient and the inlay-retained full zirconia FDP treatment planning was approved.

## 3. Preparation and Impression

**Figure 2:**
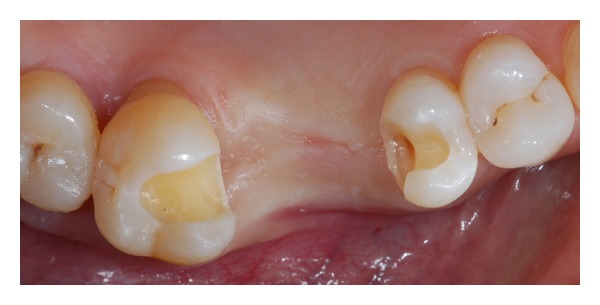
Standardized inlay preparations; previous composite fillings were removed.

The inlay preparations were designed with rounded proximal boxes and internal edges, smooth round corners, and rectangular-based preparation floors with 2.5 mm occlusal reduction, without bevels at occlusal or gingival margins. The isthmus width of the preparation was 2 mm for premolar and 3 mm for molar abutments. The minimum axial reduction (shoulder with rounded internal angle) was set at 1.5 mm and the convergence preparation angle was added up to approximately 6 degrees (Figure 2).

The minimum dimensions of the connector were 3 × 3 mm, to enhance optimum mechanical stress distribution.

Prepared dentin was sealed with an adhesive system (Scotch Bond Universal, 3M ESPE) to prevent contamination by bacteria and components coming from the impression and provisional cementation materials.

**Figure 3:**
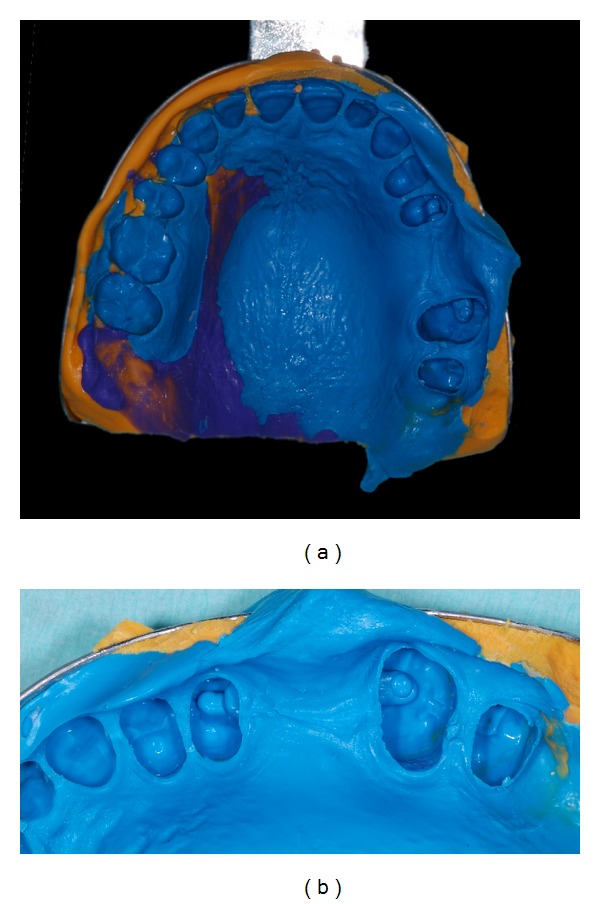
(a) Occlusal view of the final elastomeric impression. (b) Close-up of silicon impression; light body material reproduced every preparation fine details.

The impression was made using a VPS material with a one-step technique (Elite HD + putty soft, Elite HD + regular body, and Elite HD + light body, Zhermack SpA, Badia Polesine, Italy) (Figures 3(a) and 3(b)). Alginate impression of the lower arch and occlusal registration were finally performed. Inlay cavities were then filled with temporary restorations.

## 4. Try-In Fabrication

**Figure 4:**
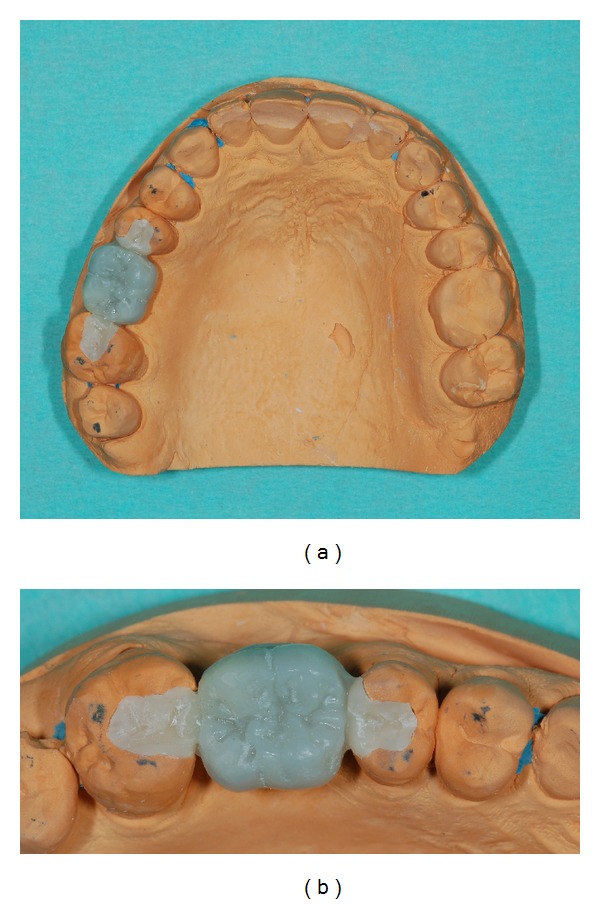
(a) Type IV gypsum master cast with the composite resin try-in of the IRFDP. (b) Close-up of the mock-up; the occlusal contacts were verified by the technician using the articulator.

Impressions were poured with Type IV gypsum (GC-Fuji Rock EP) and stone casts were mounted in an articulator. An IRFDP resin mock-up was fabricated for the clinical try-in; two different indirect laboratory light cured composite resins were used for the inlays (Sinfony, 3M ESPE) and the intermediate crown (Rigid Transparent-Blue Resin, Zirkonzahn GmbH) fabrication. Complete indirect resin photo polymerization was obtained using a laboratory curing unit (3M ESPE Alfa Light Unit) (Figures 4(a) and 4(b)).

**Figure 5:**
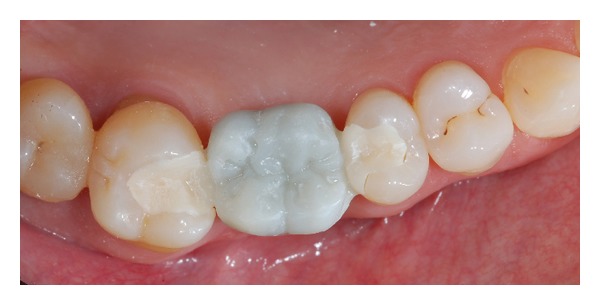
Occlusal view of the try-in seated in the oral cavity.

The fit of the structure in the oral cavity was controlled using a low-viscosity silicone material (Fit-Checker, GC, Tokyo, Japan) which demonstrated no friction and marginal integrity (Figure 5).

**Figure 6:**
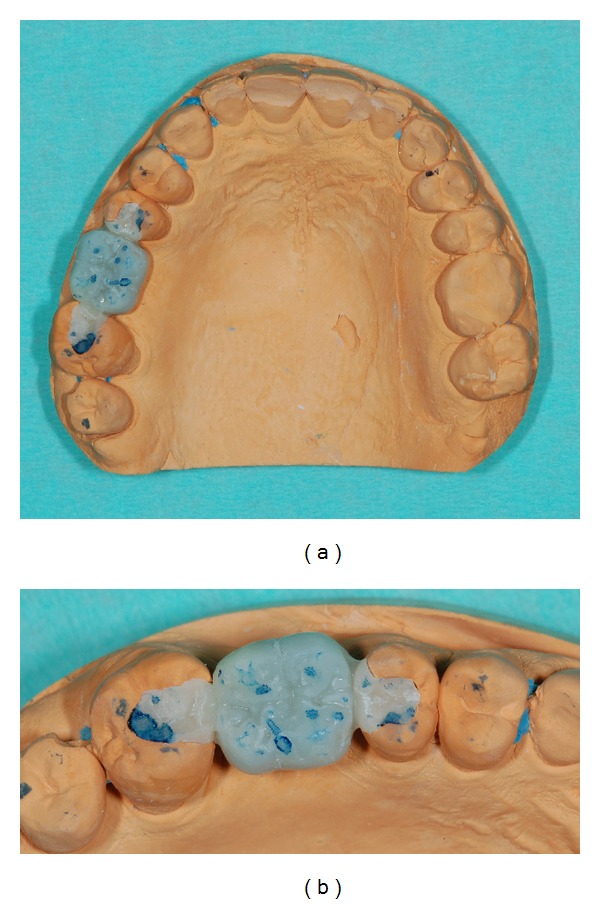
(a) Interarch relationships were highlighted with a 40 *μ* occlusal paper. (b) Details of the contacts area; excessive occlusal pressure at the margins of the restoration will be corrected in the laboratory.

The occlusion was checked with a 40 *μ*m occlusal paper (Bausch BK9, Bausch KG, Germany), both in maximum intercuspidation position and during eccentric movements, making any necessary adjustments with a fine diamond bur (Figures 6(a) and 6(b)).

## 5. CAD-CAM Procedures

The adjusted composite resin mock-up was sent to the laboratory and scanned with a fully automated optical strip-light scanner (S600 ARTI, Zirkonzahn GmbH); the lower master cast was also digitally acquired (accuracy ≤ 10 *μ*m). Interarch relationships were finally checked with a virtual articulator software to simulate occlusal movements. Presintered zirconia blank (Prettau Zirconia, Zirkonzahn GmbH) was milled with a dedicated 5 + 1 axes machine (milling unit M5, Zirkonzahn GmbH). The milled IRFDP was refinished with a tungsten carbide bur and color infiltrated with acid-free special color liquids (Colour Liquid Prettau, Zirkonzahn GmbH) using a metal-free brush.

**Figure 7:**
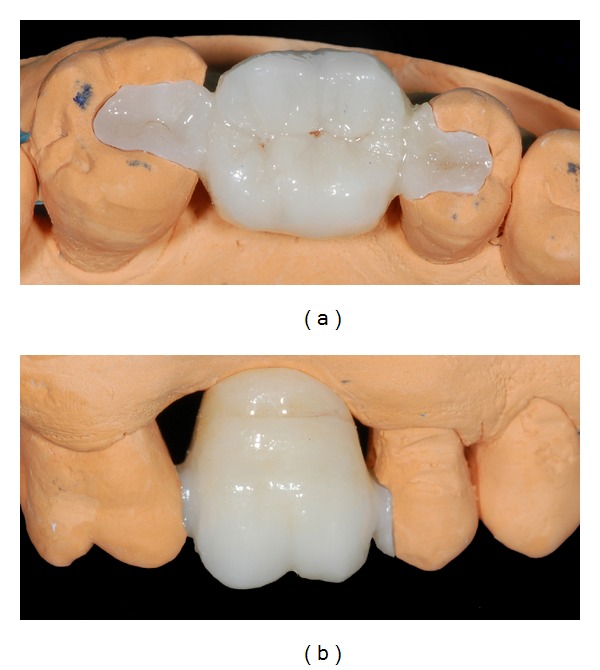
(a) Monolithic zirconia restoration on master cast: occlusal view. (b) Lateral close-up; brown stains were used in the cervical area.

After a drying stage, the sintering process was carried out in a sintering furnace (Zirkonofen 600, Zirkonzahn GmbH) until it reached 1540°C for 12 hours (Figures 7(a) and 7(b)).

## 6. Placement

The temporary restorations were removed using a manual excavator; a rubber dam was placed, isolating the preparations from the oral cavity. Abutments were cleaned using a pumice paste over a rotating brush; the cavities were treated with an intraoral sandblaster (CoJet Prep, 3M ESPE), washed out for one minute, and gently air dried. Enamel and dentin surfaces were etched for 30 s and 15 s, respectively, with 35% orthophosphoric acid and rinsed for 30 s with air/water spray. A dual-curing universal dental adhesive (ScotchBond Universal, 3M ESPE) was applied to enamel and dentin with a microbrush for 20 s, evaporated, and left uncured.

**Figure 8:**
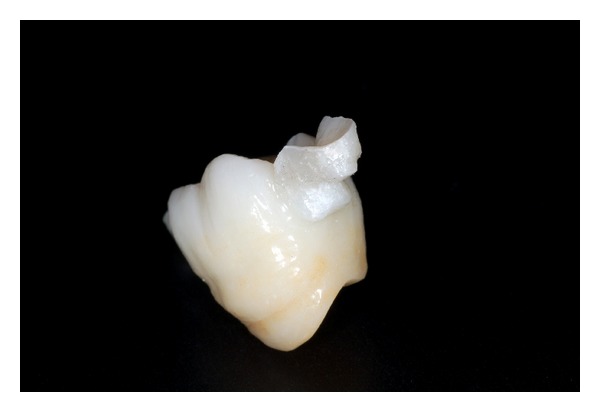
IRFDP conditioned for final adhesive cementation.

The inner side of the IRFDP was sandblasted with Al2O3 particles (50 *μ*m, 2.8 bar, 1 cm), rinsed with water spray for 60s, and ultrasonically cleaned in 95% ethyl alcohol for 10 minutes. A MDP containing primer (Clearfil Ceramic Primer, Kuraray, Japan) was applied to the zirconia surface as recommended by the manufacturer (Figure 8).

**Figure 9:**
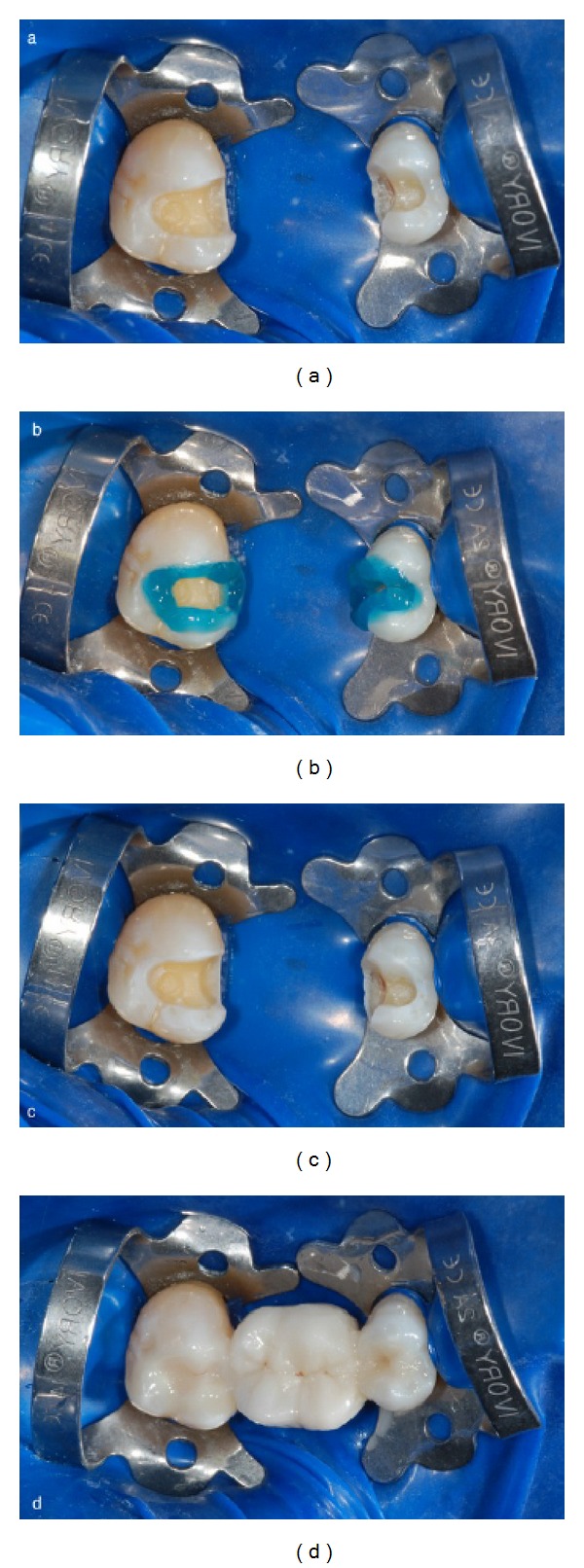
(a) Operative field isolation with dental dam. (b) Selective phosphoric acid etching. (c) Etched enamel and dentin surfaces. (d) A resin composite dual-curing cement was used.

A self-adhesive dual-curing resin cement (Panavia SA, Kuraray, Japan) was dispensed directly into the cavities using the endo tip. The solid zirconia restoration was first placed in site with a finger pressure; an ultrasonic insertion tip was used to complete the seating process, increasing the cement flow. The placement of IRFDP after adhesive procedures is resumed in Figures 9(a) to 9(d).

Excess composite resin was carefully removed using a spatula and dental floss (Oral-B Superfloss, P&G, USA). Glycerine gel was applied at the margins to prevent an oxygen inhibition layer at the interface; subsequently a prolonged light curing was performed from mesiobuccal, mesiopalatal, distobuccal, distopalatal, and occlusal directions for 90 seconds each (Bluephase LED curing light, Ivoclar). Margins were finished and polished with diamond burs, rubber points, and diamond polishing paste.

## 7. Esthetic and Functional Result

**Figure 10:**
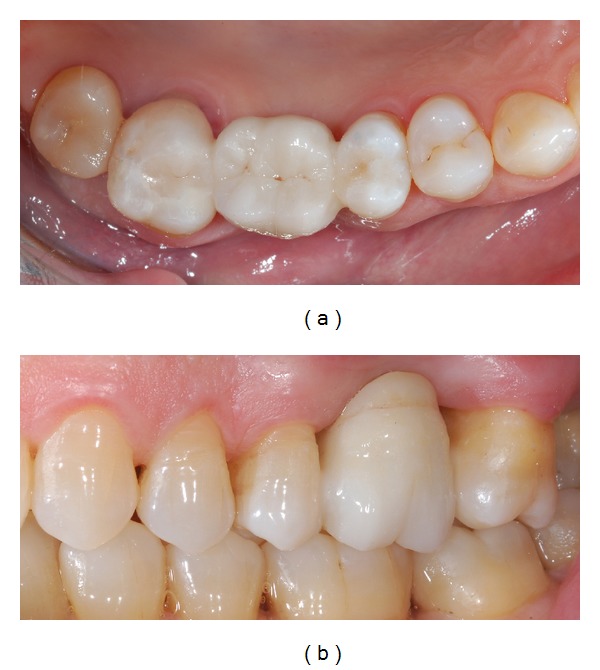
(a) Occlusal view of the luted IRFDP just after rubber dam removal. (b) Lateral view of the rehabilitation: function and esthetic were restored.

**Figure 11:**
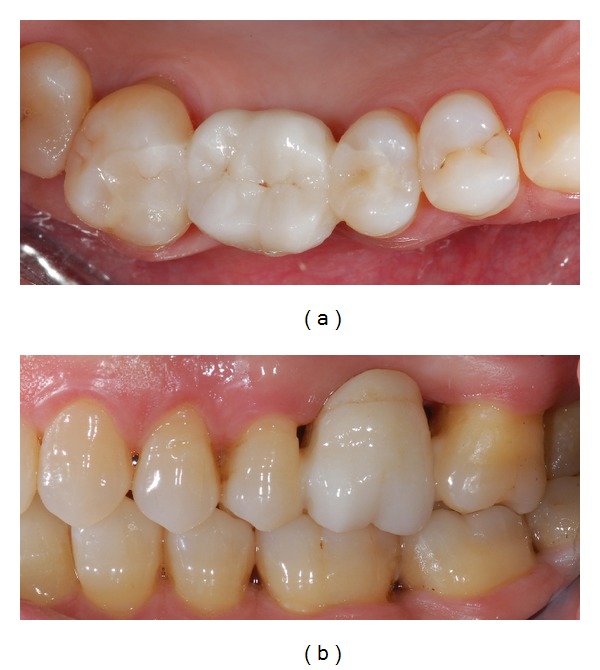
(a) 10-day follow-up: occlusal view. (b) 10-day follow-up: lateral view.

**Figure 12:**
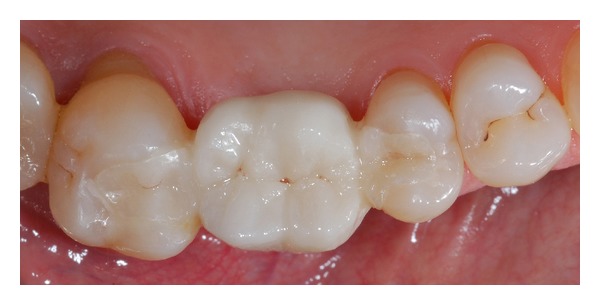
2-year follow-up: occlusal view.

Intraoral view of the luted restoration after rubber dam removal is shown in Figures 10(a) and 10(b). Nice esthetic and functional integration of the monolithic IRFDP confirms the success of the rehabilitation at 10 days (Figures 11(a) and 11(b)). Marginal integrity, absence of chipping [12]. and good gingival health status were observed at 2-year follow-up (Figure 12); the patient was also highly satisfied with the selected rehabilitation.

## 8. Discussion

It is generally accepted that partial restorations conserve sound tooth structures and are preferred over complete coverage restorations. In particular, when abutment teeth contain restorative fillings adjacent to the missing tooth, IRFDPs are considered a very minimally invasive option.

The weakest parts of IRFDPs are the connectors and the retainers; in this study a standardized inlay preparation design was used to increase the stability and retention of the densely sintered ceramic restoration [6]. Monolithic high strength ceramic FDPs demonstrated higher in vitro resistance to fracture load than metal ceramic; zirconia based materials used for IRFDPs also showed greater mechanical behavior than lithium disilicate glass-ceramic and fiber-reinforced composites [12, 19]. For zirconia IRFDP the mean fracture strength was reported to be 1248 ± 263 N when the interabutment distance was 10 mm [20].

In the last years, the demand for esthetics and biocompatibility led to the use of zirconia CAD/CAM materials in fixed prosthodontics [3]. A prospective clinical study determined the success rate of three- to four-unit posterior FDPs with Y-TZP frameworks after five years of function; the authors reported a survival rate of 85% [21]. Few studies have investigated the clinical performance of these ceramics for IRFDP rehabilitations [17, 22].

Debonding of the adhesive interface represents a common failure of the IRFDPs. The interabutment forces developed during clinical function might stress the retainer framework and luting interface; rigid connectors, with their low bending behavior, have been suggested as a possible cause of debonding [11]. Another explanation might be that inadequate bond strength values are reached between the restoration and tooth substrates. In fact, a definitive cementation protocol for high strength ceramics has not been validated yet; sandblasting of the inner side of zirconia has been suggested in the literature to increase surface roughness and promote micromechanical interlocking [18]. Different air-blasting protocols associated with chemical primers (i.e., formulations containing MDP molecules or silane coupling agents) are the most recommended conditioning methods for zirconia restorations [15, 23]; however, some studies have shown that bond strength might decrease over time due to aging of the interface and lead to failure [24].

Adequate evidences about long term safety and efficacy of solid zirconia IRFDP are required before these kinds of treatment could be recommended as acceptable for general clinical practice [6, 14].

## 9. Conclusion

Within the limits of a preliminary application, the technique described in this case report allows a minimally invasive approach for single-tooth substitution, as an alternative to a full-coverage FDP or an implant-supported crown.
